# Comparison between osteonecrosis of the humeral and femoral heads - epidemiological analysis of the surgical trend using the nationwide claims database of the republic of Korea

**DOI:** 10.1186/s12891-023-07022-4

**Published:** 2023-11-11

**Authors:** Hyeon Jang Jeong, Jung-Wee Park, Young-Kyun Lee, Kyung-Hoi Koo, Joo Han Oh

**Affiliations:** 1 Department of Orthopaedic Surgery, Seoul National University College of Medicine, Seoul National University Bundang Hospital, 82, Gumi-Ro 173 Beon-Gil, Bundang-Gu, Seongnam-Si, Gyeonggi-Do 13620 Republic of Korea; 2 Department of Orthopedic Surgery, Cheil Orthopedic Hospital, Seoul, Republic of Korea

**Keywords:** Osteonecrosis, Avascular necrosis, Surgical trends, Nationwide database

## Abstract

**Backgrounds:**

The humeral head is the second most common site of osteonecrosis, after the femoral head. However, compared to osteonecrosis of the femoral head (ONFH), epidemiological information on osteonecrosis of the humeral head (ONHH) is scarce. We hypothesised that different biomechanical properties of the shoulder from the hip joint might present different epidemiological characteristics of ONHH from those of the ONFH. To evaluate epidemiological differences, we compared trends in the surgical treatment of ONHH and ONFH using the nationwide medical claims database of the Republic of Korea (ROK).

**Methods:**

We analysed epidemiological data from the Health Insurance Review and Assessment (HIRA) database of the ROK between 2008 and 2018. HIRA database contains almost all medical information in an anonymised form, including demographics, diagnoses, and types of surgical procedures, generated through healthcare practices in ROK. The annual incidence rates of ONHH and ONFH were calculated based on the total number of the general population. Demographics, annual incidence, and the proportion of post-traumatic osteonecrosis and surgical procedures were compared according to the anatomical site and the affected year.

**Results:**

The total number of patients treated for ONHH and ONFH during the study period was 1,028 and 66,260, respectively. Although the incidence of ONHH increased, it is a relatively rare disease compared to ONFH. ONHH occurred more frequently in females, while ONFH occurred predominantly in male patients (p < 0.001). Surgical treatment for ONHH was most frequently performed in older patients (63.7%), whereas middle-aged patients had the largest proportion of ONFH (48.9%, p < 0.001). The proportion of post-traumatic osteonecrosis was significantly higher in the ONHH (5.1%) than in the ONFH (1.9%, p < 0.001). Arthroplasty was performed more frequently in the ONHH (96.0%) than in the ONFH (92.9%, p < 0.001).

**Conclusion:**

Despite the anatomical similarities between the hip and shoulder joints, the different biomechanical properties, such as weight-bearing functions, might cause epidemiological differences between ONHH and ONFH.

## Introduction

Osteonecrosis, also called avascular necrosis or bone infarction, is a degenerative bone disorder defined by the death of the bony cellular components as a result of a disruption in the subchondral blood supply [1]. The humeral head is the second most common site of osteonecrosis after the femoral head [2]. However, information regarding osteonecrosis of the humeral head (ONHH) is relatively scarce compared to that regarding osteonecrosis of the femoral head (ONFH). Several previous studies have reported the annual incidence of ONFH [3, 4], whereas the annual incidence of ONHH using a nationwide survey has not been reported to the best of the author’s knowledge.

Several previous studies have reported various causes of ONHH and ONFH, such as trauma, genetic predispositions, use of steroids, and excessive alcohol consumption [5–7]. These factors can damage the vascular endothelial tissue and induce microvascular thrombosis [8–10]. Furthermore, pharmacologic factors including steroids and alcohol affect the metabolism of bone marrow stromal cells and induce intramedullary adipogenesis, which increase intraosseous pressure [11–13]. Arterial occlusion and venous stasis, leading to the death of the cellular components of the bone, can be induced by increased intraosseous pressure [11]. Furthermore, several studies have attempted to elucidate the underlying pathomechanism correlated to microRNAs that regulate the differentiation of bone marrow mesenchymal stem cells into adipogenic progenitor cells [14, 15]. Conventionally, steroid use and excessive alcohol consumption have been reported as the most common risk factors of ONFH [5, 6]. Meanwhile, although it has been speculated that ONHH is associated with trauma or post-traumatic sequelae [7, 16], there is little evidence to support this claim.

The natural history of osteonecrosis is varied according to the size and site of disease occurrence [17]. However, in most cases, osteonecrosis is a progressive disease that results in a collapse of the bony structure if not treated appropriately [1]. To treat ONHH and ONFH, several procedures, including arthroplasty and joint-preserving procedures, have been performed. Although favourable outcomes have been reported for joint-preserving procedures in low-grade ONHH [18, 19], studies with a high level of evidence for the treatment of ONHH are relatively scarce compared to those for ONFH. Furthermore, the shoulder is a non-weight-bearing joint, in contrast to the hip, and this anatomical and functional difference might present as differences in the distribution of factors such as age at operation, sex, and preferred surgical treatment methods. We hypothesised that different biomechanical properties of the shoulder from the hip joint might present different epidemiological characteristics of ONHH from those of the ONFH. Therefore, in this study, we aimed to evaluate epidemiological differences by comparing the trend of surgical treatment for ONHH and ONFH using the nationwide medical claims database of the Republic of Korea (ROK).

## Methods

The Health Insurance Review and Assessment (HIRA) database of the ROK was analysed in this study. Approximately 97% of citizens in the ROK are covered by the Korean National Health Insurance Program (KNHIP), and the remaining 3% are supported by the ROK government through a medical aid programme. Both the KNHIP and medical aid programmes submit medical data to the HIRA for reimbursement. Therefore, almost all healthcare practices are reviewed by the HIRA, and medical information, including demographics, diagnoses, procedures, and prescriptions, can be accessed in an anonymised form. This medical information, which is stratified by the Korean Classification of diseases-8 (KCD-8) based on the International Classification of disease-10 (ICD-10) and electronic data interchange (EDI) codes, is accessible through the HIRA database.

We extracted the medical data of patients admitted due to osteonecrosis of the humeral or femoral head between January 2008 and December 2018. ONHH was defined using the following KCD-8 codes: M8701, M8711, M8721, M8731, M8781, M9031, M9041, and M9051. ONFH was also classified using the following KCD-8 codes: M8705, M8715, M8725, M8735, M8785, M9035, M9045, and M9055. Osteonecrosis was divided into traumatic (M8721, M8725 [M872x]) and non-traumatic causes to minimise errors based on incorrect coding. Patients’ chronological age is reported in 10-year increments in the HIRA database, and we stratified them into three groups: young (age < 40 years), middle-aged (age between 40 and 59 years), and older (age ≥ 60 years) adults. Patients younger than 20 years were excluded from the analysis because of the rare incidence of ONFH in that population [3, 4].

Procedural codes to treat ONHH or ONFH were analysed from the extracted data. Unlike the hip joint, there are two types of total shoulder replacement arthroplasty; anatomical total shoulder arthroplasty (aTSA) which mimics the native shoulder joint, and reverse total shoulder arthroplasty (rTSA) which focuses on functional restoration of the shoulder joint by replacing the function of the rotator cuffs by the deltoid muscle. However, as the procedural code in the HIRA database cannot distinguish aTSA from rTSA, we simplified the procedural code for the total hip replacement arthroplasty, aTSA, and rTSA as total hip/shoulder replacement arthroplasty. Therefore, the procedural codes in this study were classified as total hip/shoulder replacement arthroplasty (TRA), hemiarthroplasty (HA), core decompression and/or multiple drilling (CD), vascularised fibular graft (VFG), and corrective osteotomy (CO). The annual incidence was calculated as the number of each procedure/100,000 person-years using the total number of each procedure and the total number of people equal to or older than 20 years of age, taken from the annual population data of ROK as recorded by Statistics Korea, the central administrative agency of the ROK.

All statistical analyses were conducted using R version 4.0.5 (The R Foundation for Statistical Computing, Vienna, Austria) and RStudio version 1.4.1106 (RStudio Inc., Boston, MA, US). Chi-square or Fisher’s exact tests were conducted to compare proportional differences between various groups. A generalised linear model using Poisson regression was used to compare the annual incidence during the study period. All statistical analyses were performed on both sides, and the significance level was set at p < 0.05.

## Results

The total number of patients treated for ONHH and ONFH during the study period was 1,028 and 64,261, respectively. ONHH occurred more frequently in women (61.8%) than men (38.2%); however, the prevalence of ONFH was higher in men (68.8%) than in women (31.2%). There was a significant gender difference between the two groups (p < 0.001, Table 1). Post-traumatic osteonecrosis occurred more frequently in the ONHH group (5.1%) than in the ONFH group (1.9%, p < 0.001).

**Table 1:** Sex and age group of patients who underwent surgery for ONHH or ONFH between 2008 and 2018

	ONHH	ONFH	P value
**Total**	1,028 (100%)	64,261 (100%)	
**Sex**			
Male	393 (28.2%)	44,206 (68.8%)	< 0.001^*^
Female	635 (61.8%)	20,055 (31.2%)	
**Age group**			
Young	57 (5.6%)	8,633 (13.4%)	< 0.001^*^
20–29	12 (1.2%)	1,884 (2.9%)	
30–39	45 (4.4%)	6,749 (10.5%)	
Middle-aged	314 (30.7%)	31,413 (48.9%)	
40–49	119 (11.6%)	12,978 (20.2%)	
50–59	195 (19.0%)	18,435 (28.7%)	
Older adult	657 (63.9%)	24,215 (37.7%)	
60–69	287 (27.9%)	13,808 (21.5%)	
70–79	315 (30.6%)	8,712 (13.6%)	
≥ 80	55 (5.4%)	1,695 (2.6%)	

Data are presented as the number of patients (proportion)

ONHH: osteonecrosis of the humeral head, ONFH: osteonecrosis of the femoral head

*statistically significant

**Table 2:** Proportion and annual incidence rate of patients who underwent surgery for ONHH and ONFH between 2008 and 2018

Year	TRA	HA	CO	VFG	CD	Total
**ONHH**	524 (51.0%)	463 (45.0%)	6 (0.6%)	0 (0.0%)	35 (3.4%)	1,028
2008	9 (19.1%)	34 (72.3%)	0 (0.0%)	0 (0.0%)	4 (8.5%)	47 (0.13)
2009	11 (22.4%)	37 (75.5%)	1 (2.0%)	0 (0.0%)	0 (0.0%)	49 (0.13)
2010	28 (36.8%)	43 (56.6%)	0 (0.0%)	0 (0.0%)	5 (6.6%)	76 (0.20)
2011	29 (36.7%)	48 (60.8%)	0 (0.0%)	0 (0.0%)	2 (2.5%)	79 (0.20)
2012	39 (38.6%)	59 (58.4%)	0 (0.0%)	0 (0.0%)	3 (3.0%)	101 (0.26)
2013	40 (42.1%)	51 (53.7%)	1 (1.1%)	0 (0.0%)	3 (3.2%)	95 (0.24)
2014	55 (57.3%)	39 (40.6%)	0 (0.0%)	0 (0.0%)	2 (2.1%)	96 (0.24)
2015	63 (55.3%)	42 (36.8%)	3 (2.6%)	0 (0.0%)	6 (5.3%)	114 (0.27)
2016	77 (63.6%)	38 (31.4%)	0 (0.0%)	0 (0.0%)	6 (5.0%)	121 (0.30)
2017	71 (62.8%)	40 (35.4%)	0 (0.0%)	0 (0.0%)	2 (1.8%)	113 (0.27)
2018	102 (74.5%)	32 (23.4%)	1 (0.7%)	0 (0.0%)	2 (1.5%)	137 (0.33)
**ONFH**	55,417 (86.2%)	4,252 (6.6%)	171 (0.3%)	62 (0.1%)	4,359 (6.8%)	64,261
2008	3,516 (81.3%)	400 (9.3%)	40 (0.9%)	9 (0.2%)	358 (8.3%)	4,323 (11.56)
2009	3,829 (83.6%)	364 (7.9%)	19 (0.4%)	7 (0.2%)	360 (7.9%)	4,579 (12.10)
2010	4,094 (83.3%)	425 (8.6%)	17 (0.3%)	9 (0.2%)	369 (7.5%)	4,914 (12.85)
2011	4,473 (83.8%)	378 (7.1%)	19 (0.4%)	5 (0.1%)	461 (8.6%)	5,336 (13.80)
2012	4,736 (83.1%)	452 (7.9%)	15 (0.3%)	5 (0.1%)	489 (8.6%)	5,697 (14.56)
2013	5,198 (85.0%)	430 (7.0%)	11 (0.2%)	3 (0.0%)	474 (7.8%)	6,116 (15.45)
2014	5,194 (85.7%)	369 (6.1%)	14 (0.2%)	9 (0.1%)	477 (7.9%)	6,063 (15.13)
2015	5,612 (87.4%)	408 (6.4%)	9 (0.1%)	4 (0.1%)	386 (6.0%)	6,419 (15.16)
2016	6,106 (89.4%)	346 (5.1%)	11 (0.2%)	8 (0.1%)	359 (5.3%)	6,830 (16.68)
2017	6,354 (90.1%)	374 (5.3%)	6 (0.1%)	3 (0.0%)	318 (4.5%)	7,055 (17.06)
2018	6,305 (91.0%)	306 (4.4%)	10 (0.1%)	0 (0.0%)	308 (4.4%)	6,929 (16.61)

Data are presented as the number of patients (proportion) or the number of patients (annual incidence rate: 100,000 person-years)

TRA, total replacement arthroplasty; HA, hemiarthroplasty; CO, corrective osteotomy; VFG, vascularised fibular graft; CD, core decompression; ONHH, osteonecrosis of the humeral head; ONFH, osteonecrosis of the femoral head

The annual incidence of ONHH in 2008 was 0.13/100,000 person-years. In 2018, the annual incidence was 0.33/100,000 person-years, which nearly tripled (p < 0.001, Table 2). Similarly, the annual incidence of ONFH increased from 11.56/100,000 person-years to 16.61/100,000 person-years during the study period (p < 0.001, Table 2).

Surgical treatment for ONHH was most frequently performed in the older patient group (63.9%), while middle-aged patients had the largest proportion of ONFH (48.9%, p < 0.001, Table 1). Arthroplasty was performed more frequently in the ONHH group (96.0%) than in the ONFH group (92.9%, p < 0.001). The TRA was the most frequently used procedure to treat osteonecrosis in both the ONHH and ONFH groups; however, the proportion of procedures was significantly different (p < 0.001, Table 2). In the ONHH group, the proportion of TRA rapidly increased from 19.1% to 2008 to 74.5% in 2018 (p < 0.001, Table 2). However, the proportions of HA (p = 0.140), CO (p = 0.422), and CD (p = 0.838) did not change significantly, and VFG was not performed during the study period (Table 2). In the treatment of ONFH, the proportion of patients who underwent TRA during the study period also increased (p < 0.001); however, the proportions of HA (p < 0.001), CO (p < 0.001), VFG (p = 0.014), and CD (p = 0.003) decreased during the same period (Table 2).

**Fig. 1:**
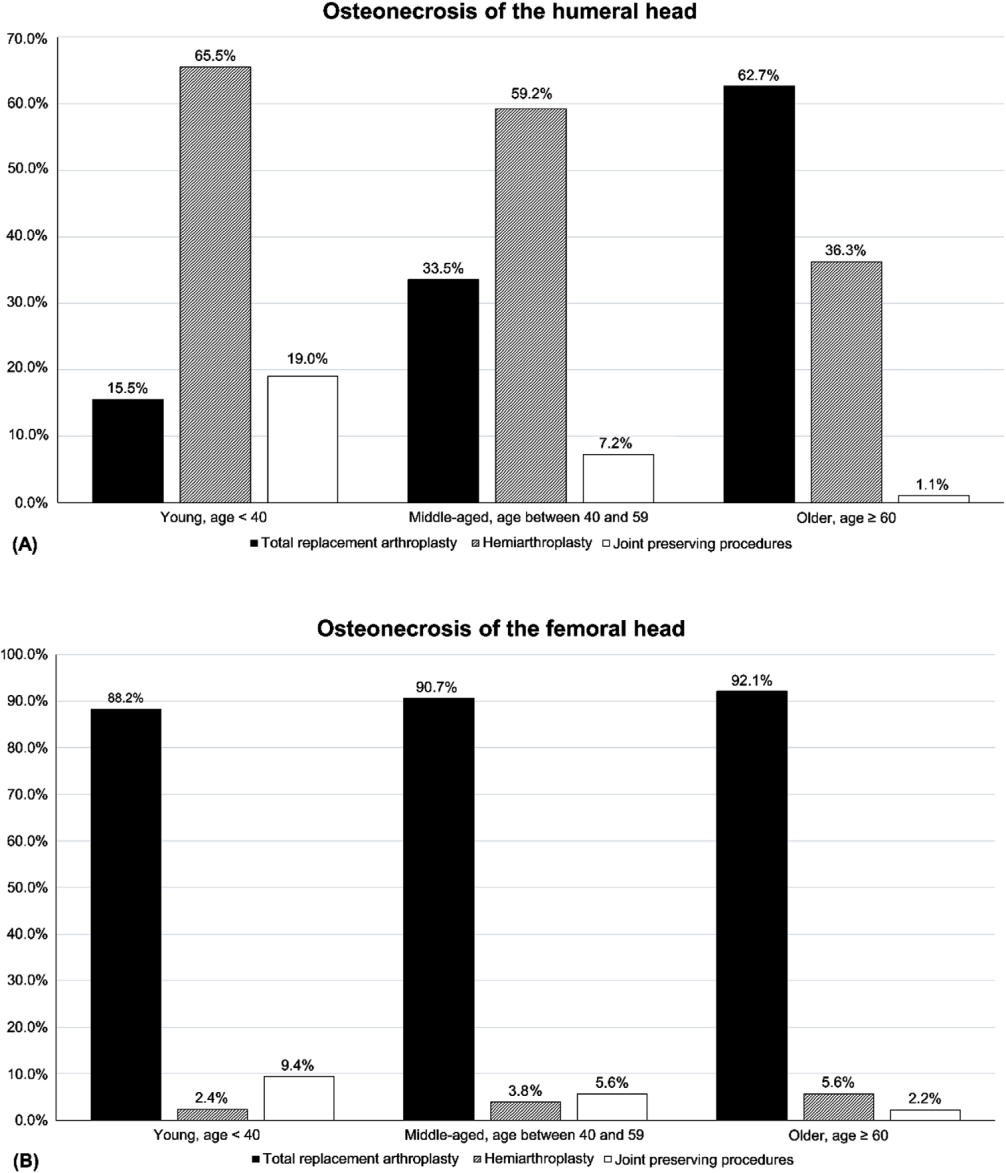
The proportion of surgical procedures according to age. (**A**) In the humeral head, total replacement arthroplasty was preferred in older patients, while hemiarthroplasty and joint-preserving procedures were preferred in younger patients. (**B**) In the femoral head, total replacement arthroplasty was preferred for all ages. The percentage of surgical procedures according to age group was significantly different between the humeral head and femoral head (p < 0.01)

In both ONHH and ONFH groups, joint preserving procedures including CD, VFG, and CO have been conducted in younger patient group more frequently (Fig. 1). In the ONHH group, the older patients group has been treated with TRA most frequently, whereas the young and middle-aged patients group has been treated with HA most frequently (Fig. 1). However, in the ONFH group, TRA has been most frequently conducted regardless of age group (Fig. 1). Preferred operation methods according to the age group were significantly different between the osteonecrosis of the humeral and femoral heads (p < 0.001).

## Discussion

Our study showed that, although the prevalence of ONHH increased during the study period, it is a relatively rare disease compared to ONFH. ONHH occurred more frequently in women, and the mean age at surgery was higher than that of patients with ONFH. Furthermore, the proportion of post-traumatic osteonecrosis was significantly higher in the ONHH group than in the ONFH group. Although joint preserving procedures have been more frequently attempted in the younger patient group than in the middle-aged and older patient groups in both ONHH and ONFH groups, arthroplasties including TRA and HA accounted for the majority of surgical treatments in all age groups. The age at operation and the proportion of arthroplasty were significantly higher in the ONHH group than in the ONFH group.

In this study, the annual incidence of ONHH was significantly lower than that of ONFH, and the ONHH prevalence in the age group was also higher than that in the ONFH group. Both the shoulder and hip joints have anatomical similarities as they are proximal ball-and-socket joints; however, in terms of weight-bearing, there is a fundamental functional difference. The load on the hip joint during running has been reported to be up to 10 times the body weight [20], while the load on the shoulder joint does not exceed the body weight in most activities of daily life [21]. Weight-bearing increases the joint reaction forces, and the broad spectrum of the disease might be affected by the increased load on the joint [22]. Although we could not investigate the detailed medical information, including the time of onset of osteonecrosis, we presumed that the presence and/or aggravation of pain according to weight-bearing might be one of the reasons for the difference in age distribution between ONHH and ONFH. Relatively less load on the shoulder joint due to its non-weight-bearing nature might be a cause for a longer tolerance for pain before surgical treatment, which leads to an older patient group with more progressive osteonecrosis.

The difference in sex predominance between the two groups might be another issue. Osteonecrosis is a multifactorial disease, and we do not know exactly why female predominance is observed in ONHH. However, female predominance in the incidence of osteoporotic proximal humeral fractures might be one of the reasons for this [23–25]. Rates of post-traumatic ONHH have been reported to range from 1 to 10% [26, 27]. As is well known, the vascular supply of the humeral head originates from the anterior and posterior humeral circumflex branches of the axillary artery [28], and the displacement of the medial humeral calcar could compromise vascular supply to the humeral head [29]. Therefore, the complexity of the proximal humeral fracture [30] and the anatomical site of fracture involvement [29] were considered risk factors for post-traumatic ONHH. Proximal humeral fractures occur more frequently in old, female patients [23, 25], and Bahrs et al. reported that most patients with more complex fractures were female patients older than 60 years [24].

Osteonecrosis after arthroscopic rotator cuff repair could also be another reason for the female predominance in ONHH [31]. Keough et al. recently reviewed several case series regarding osteonecrosis after arthroscopic rotator cuff repair [31]. They argued that vascular injury of the anterior circumflex humeral artery during rotator cuff repair might be linked to the postoperative osteonecrosis of the humeral head. Although they have not suggested the reason, their reviewed case series presented female predominance in postoperative ONHH.

KCD-8 subclassified the aetiology of osteonecrosis; however, most aetiologies were idiopathic (M870x) or unclassified/unidentified (M873x, M878x, M879x, and M905x). Secondary osteonecrosis with specific causes (M903x, M904x) is extremely rare, occurring in caisson disease or haemoglobinopathy. Furthermore, KCD-8 simply classifies osteonecrosis due to drugs (M871x) rather than distinguishing between steroid- and alcohol-induced osteonecrosis. Therefore, we simplified the aetiologies of osteonecrosis as traumatic or non-traumatic to minimise errors based on inaccurate categorisation.

In the present study, the proportion of patients with post-traumatic ONHH was significantly greater than that of those with ONFH. Although several previous studies have argued that ONHH is associated with trauma or post-traumatic sequelae [7, 16], epidemiological information regarding the aetiology of ONHH is scarce. Although the data analysed in this study did not contain detailed information on comorbidities, alcohol consumption history, and/or history of steroid use, we considered this study to be clinically important as it elucidated that the proportion of post-traumatic osteonecrosis is relatively higher in the humeral head than in the femoral head using a nationwide database that included data from almost all healthcare practices.

In both ONHH and ONFH, the proportion of arthroplasty was higher than that of joint-preserving procedures, and TRA was the most frequently performed procedure in both groups. Interestingly, the proportion of TRA in ONHH rapidly increased from 19.1% to 2008 to 74.5% in 2018. Theoretically, HA is sufficient for treating ONHH without glenoid erosion and/or rotator cuff deficiency [32]. We considered that the prevalent age group of surgical treatment for ONHH might be one of the reasons for the higher proportion of TRA in ONHH. The prevalence of ONHH was significantly higher than that of ONFH. Furthermore, previous studies have reported that concomitant osteonecrosis of other joints was found in 75–90% of atraumatic ONHH [33–35], whereas concomitant ONHH was found in 20% of ONFH [36]. Therefore, we estimated that the biomechanical properties of the glenohumeral joint could endure a longer tolerance period for pain in ONHH. However, ONHH is a progressive disease that cannot be reversed, and a prolonged tolerance period can aggravate the deformity of the humeral head, which induces degenerative arthritic changes in the glenoid.

The introduction of rTSA might be another reason for the dramatic increase in TRA procedures in ONHH treatment. Although the procedural code in the HIRA database cannot distinguish aTSA from rTSA, we estimated that the increasing trend of TRA was closely related to the increased use of rTSA. rTSA has the advantage of decreased incidence of loosening of the glenoid implant, which is one of the most common complications of aTSA [37], and can be used in patients with rotator cuff deficiency. It continues to expand indications from irreparable rotator cuff tear and cuff tear arthropathy to complex fractures [38], osteoarthritis [39], and ONHH [40].

The clinical prevalence of ONHH is very rare. However, as post-traumatic osteonecrosis more frequently occurs in the humeral head than in the femoral head, ONHH prevalence might be decreased by the prevention and/or early treatment of post-traumatic ONHH, especially in high-risk fracture patterns with a short medial hinge on the head fragment, disrupted integrity of the medial hinge from the adjacent bone, or capsular detachment from the humeral head [41, 42].

Although the time interval between the proximal humeral fracture and the ONHH could not be assessed in this study, it has been reported that ONHH has been diagnosed on average 11.8 months after the operation [43], and ONHH incidence increased with time progression, despite stable plate fixation [44]. However, joint preserving procedures including CD presented favourable outcomes in the early stages of osteonecrosis [45–47], and these procedures could be combined with regenerative medicines such as platelet-rich plasma and mesenchymal stem cells [46]. Therefore, considering that the onset of ONHH-related symptoms might be delayed because of the biomechanical properties of the shoulder joint, the periodic monitoring of ONHH should be mandatory, especially in high-risk patients.

To the best of our knowledge, this is the first study to evaluate the epidemiology of ONHH using a nationwide database survey and comparing it with that of ONFH. However, it has inherent limitations that accompany a large database study. The analysed data were extracted from anonymised databases. As detailed medical information was omitted, the possibility of erroneous coding that could violate the accuracy of the database could not be evaluated. However, this large database covered nearly 100% of all surgical procedures on ONHH and ONFH in the ROK during the study period, in accordance with the laws of the ROK. Therefore, we considered the statistical power of this database acceptable. Additionally, we only analysed the surgical trend of osteonecrosis, not including the non-surgical treatments including medication and/or rehabilitation. However, current non-surgical treatment options are focused on symptom relief and cannot prevent irreversible changes. Additionally, we could not assess the outcomes of these treatment options in the HIRA database because of the absence of detailed medical information. Therefore, we considered that the analyses of the surgical trend might be more appropriate to represent the epidemiological characteristics of osteonecrosis. Finally, this study has the limitation of not being able to clearly determine the time interval between disease occurrence and surgical treatment. Thus, the treatment approach might have limitations in clinical applications. To overcome this limitation, further research including prospective cohort studies might be needed.

## Conclusion

Despite the anatomical similarities between the hip and shoulder joints, the different biomechanical properties according to weight bearing might cause epidemiological differences between ONHH and ONFH.

## Data Availability

The data presented in this study are available on request from the corresponding author. The data are not publicly available due to regulation of the Health Insurance Review and Assessment of the Republic of Korea.

## List of Abbreviations

ONHH: Osteonecrosis of the humeral head
ONFH: Osteonecrosis of the femoral head
ROK: The Republic of Korea
HIRA: The Health Insurance Review and Assessment of the Republic of Korea
KNHIP: The Korean National Health Insurance Program
KCD-8: The Korean Classification of diseases-8
ICD-10: The International Classification of disease-10
EDI: Electronic data interchange codes
TRA: Total shoulder or hip replacement arthroplasty
HA: Hemiarthroplasty
CD: Core decompression and/or multiple drilling
VFG: Vascularised fibular graft
CO: Corrective osteotomy
